# Genome wide association study of the whiteness and colour related traits of flour and dough sheets in common wheat

**DOI:** 10.1038/s41598-021-88241-4

**Published:** 2021-04-22

**Authors:** Mengqi Ji, Wenqi Fang, Wenshu Li, Yunzhe Zhao, Yingxin Guo, Wei Wang, Guangfeng Chen, Jichun Tian, Zhiying Deng

**Affiliations:** 1 State Key Laboratory of Crop Biology, Key Laboratory of Crop Biology of Shandong Province, Group of Wheat Quality and Molecular Breeding, College of Agronomy, Shandong Agricultural University, Tai’an, 271000 Shandong People’s Republic of China; 2 College of Ecology and Garden Architecture, Dezhou University, Dezhou, 253023 Shandong People’s Republic of China

**Keywords:** Genetic markers, Genomics, Plant breeding, Plant breeding

## Abstract

Flour whiteness and colour are important factors that influence the quality of wheat flour and end-use products. In this study, a genome wide association study focusing on flour and dough sheet colour using a high density genetic map constructed with 90K single nucleotide polymorphism arrays in a panel of 205 elite winter wheat accessions was conducted in two different locations in 2 years. Eighty-six significant marker-trait associations (MTAs) were detected for flour whiteness and the brightness index (L* value), the redness index (a* value), and the yellowness index (b* value) of flour and dough sheets (P < 10^–4^) on homologous group 1, 2, 5 and 7, and chromosomes 3A, 3B, 4A, 6A and 6B. Four, three, eleven, eleven MTAs for the flour whiteness, L* value, a* value, b* value, and one MTA for the dough sheet L* value were identified in more than one environment. Based on MATs, some important new candidate genes were identified. Of these, two candidate genes, *TraesCS5D01G004300* and *Gsp-1D,* for BS00000020_51 were found in wheat, relating to grain hardness. Other candidate genes were associated with proteins, the fatty acid biosynthetic process, the ketone body biosynthetic process, etc.

## Introduction

Wheat (*Triticum aestivum* L.) is one of the most important food crops worldwide, and it is also the source of major cooking foods in China. With the improvement of people’s living standards, the requirements for wheat colour have also increased significantly. Moreover, flour colour significantly influences the quality of wheat end-use products^1,14^. Therefore, it is necessary to pay attention to the study of flour colour to improve the quality of wheat products to meet the development needs of the market.

Flour colour and whiteness are important indexes of wheat flour quality that can reflect flour quality and milling precision and are also important indicators of flour grading. Flour colour (whiteness) is a quantitative trait controlled by multiple genes with high heritability^2–4^. Some researchers found that the flour brightness index (L* value), the redness index (a* value), and the yellowness index (b* value) could be used to evaluate flour colour^5–7^. In 2002, the CIE-L*, a*, b* colour system was introduced for the colour determination of Chinese flour and noodles^8^.

Although there have been many studies on the quantitative trait locus (QTL) mapping of wheat flour quality-related traits^9,10^, genetic dissection of the flour and dough sheet colours was rarely conducted at the same time. Through QTL mapping, some QTLs that control the stability of noodle colour traits were found on chromosomes 2A and 2D using DH populations^11^. The major QTLs/genes that affect the brightness of wheat flour were also detected on chromosomes 4D, 4A and 5D, ranging from 10 to 23.4% of PVE, and some QTLs associated with the yellowness of wheat flour were detected on chromosomes 7A and 4D^12^. However, for flour colour-related traits, the genotype rather than the environment was the main determinant^13^. In addition, the dough sheet colour is also important for noodle quality. The sheet colour score accounts for 9–45% of the total score when evaluating noodle quality^14^. With respect to dough sheet colour, the L* value is significantly affected by the protein content, and the a* value is highly correlated with the yellow pigment content. However, with respect to fresh dough sheets, PPO (polyphenol oxidase) activity significantly decreases the L* value, and highly significant positive correlations between the a* and b* values of fresh dough colour and PPO activity were reported from 4 to 24 h under resting conditions^15^. However, there is little information on the genetic dissection of the colour of dough sheets.

A genome-wide association study (GWAS) is a powerful approach for mapping economically and biologically valuable traits of germplasm collections. This method has great potential in determining the locus responsible for a specific phenotype and for analysing the complex traits of crops^16^. Recently, GWAS has been widely used to study the complex traits of various plant species, such as *Arabidopsis*^17,18^, rice^19,20^, corn^21–23^ and durum wheat^24^. Although this method has also been used in wheat^25–29^, genome-wide association mapping of the colour-related flour and dough sheet traits has rarely been reported.

Therefore, the present study used GWAS to dissect the whiteness and colour-related traits of flour and dough sheets using 24,355 single nucleotide polymorphisms (SNPs) genotyped using the 90K Illumina iSelect array in a population of diverse winter wheat varieties. The objective of this study was to identify markers and candidate genes for loci associated with these traits to improve wheat flour and dough colour quality by breeding.

## Results

### Phenotypic variation in colour traits of flour and dough sheets

The phenotypic values for flour whiteness, flour colour and dough sheet colour within four environments (TA 2015, DZ 2015, TA 2016, DZ 2016) are shown in Tables 1 and 2. Extensive phenotypic variation for these traits was observed across four environments (i.e., two growing seasons and two locations) among the 205 winter wheat accessions. The whiteness, L*, a* and b* values of the flour and dough sheets were continuously distributed in the population (Fig. S1 and Fig. S2), which like typical quantitative traits, indicating that they were genetically controlled by multiple genes. Analysis of variance showed significant differences in the flour whiteness and L*, a* and b* values of the flour and dough sheets (P < 0.0001) among genotypes and environments, as well as G × E interactions (Table S1). The *h*^2^ values of flour whiteness and the b* value were 98% and 99%, respectively (Table 1). The *h*^2^ values of the a* and b* values of the fresh dough sheets (FDS) and the b* value of the dry dough sheets (DDS) were 50%, 48% and 46%, respectively (Table 2).

**Table 1:** Phenotypic analysis of flour whiteness and colour in two locations in 2 years.

Trait	Environment	Minimum	Maximum	Mean	SD	Skewness	*h*^2^ (%)
Whiteness	TA 2015	68.20	80.30	73.93	2.56	0.50	98.5
	DZ 2015	68.30	82.10	74.58	2.59	0.66	
	TA 2016	68.70	80.90	75.01	2.25	0.34	
	DZ 2016	69.20	82.60	75.69	2.27	0.68	
L* value	TA 2015	69.71	74.6	72.24	0.77	0.14	24.2
	DZ 2015	67.48	74.13	72.37	0.80	− 1.04	
	TA 2016	72.58	75.69	73.84	0.55	0.46	
	DZ 2016	72.36	75.36	73.74	0.54	0.40	
a* value	TA 2015	− 2.93	− 0.68	− 1.63	0.40	− 0.27	–
	DZ 2015	− 2.42	− 0.32	− 1.35	0.35	− 0.30	
	TA 2016	− 1.67	− 0.32	− 0.85	0.26	− 0.29	
	DZ 2016	− 1.99	− 0.68	− 0.66	0.29	− 0.27	
b* value	TA 2015	3.87	10.53	7.53	1.37	− 0.32	99.1
	DZ 2015	3.51	11.31	7.26	1.32	− 0.31	
	TA 2016	4.83	10.80	7.60	1.07	0.12	
	DZ 2016	4.32	10.41	7.08	1.01	− 0.05	

*TA* Tai’an location, *DZ* Dezhou location, L* value the brightness index, a* value the redness index, b* value the yellowness index.

**Table 2:** Phenotypic analysis of fresh and dry dough sheet colour in two locations in 2 years.

	Trait	Environment	Minimum	Maximum	Mean	SD	Skewness	*h*^2^ (%)
FDS colour	L* value	TA 2015	75.76	91.79	86.15	3.00	− 0.67	28.9
		DZ 2015	61.91	96.80	83.09	4.17	− 1.40	
		TA 2016	64.99	89.16	84.27	3.37	− 2.27	
		DZ 2016	61.91	93.39	83.08	4.25	− 2.07	
	a* value	TA 2015	− 3.47	− 0.09	− 1.43	0.54	− 0.07	50.0
		DZ 2015	− 3.66	− 0.53	− 1.77	0.83	0.11	
		TA 2016	− 3.80	− 0.04	− 1.96	0.80	0.29	
		DZ 2016	− 3.89	− 0.53	− 1.59	0.65	0.23	
	b* value	TA 2015	8.18	21.31	12.94	2.30	0.85	48.1
		DZ 2015	8.52	24.72	15.98	2.57	0.41	
		TA 2016	8.52	27.09	17.76	2.82	0.25	
		DZ 2016	11.08	24.70	17.10	2.63	0.38	
DDS colour	L* value	TA 2015	64.44	89.39	80.59	4.74	− 0.82	–
		DZ 2015	55.85	88.25	75.42	5.51	− 0.46	
		TA 2016	61.13	87.47	76.69	5.03	− 0.62	
		DZ 2016	58.69	86.12	75.56	4.84	− 0.72	
	a* value	TA 2015	− 2.81	0.00	− 0.64	0.47	− 1.22	–
		DZ 2015	− 3.19	− 0.53	− 0.97	0.62	− 0.66	
		TA 2016	− 8.00	− 0.53	− 1.07	0.80	− 3.30	
		DZ 2016	− 2.43	− 0.53	− 1.04	0.64	− 0.10	
	b* value	TA 2015	8.52	26.76	14.71	2.75	0.88	46.0
		DZ 2015	8.52	23.60	16.42	2.34	0.30	
		TA 2016	8.52	28.15	17.64	2.73	0.33	
		DZ 2016	11.96	24.71	16.96	1.98	0.29	

*TA* Tai’an location, *DZ* Dezhou location, L* value the brightness index, a* value the redness index, b* value the yellowness index, *FDS* fresh dough sheet, *DDS* dry dough sheet.

### Marker–trait associations (MTAs) of colour traits of flour

**Figure 1:**
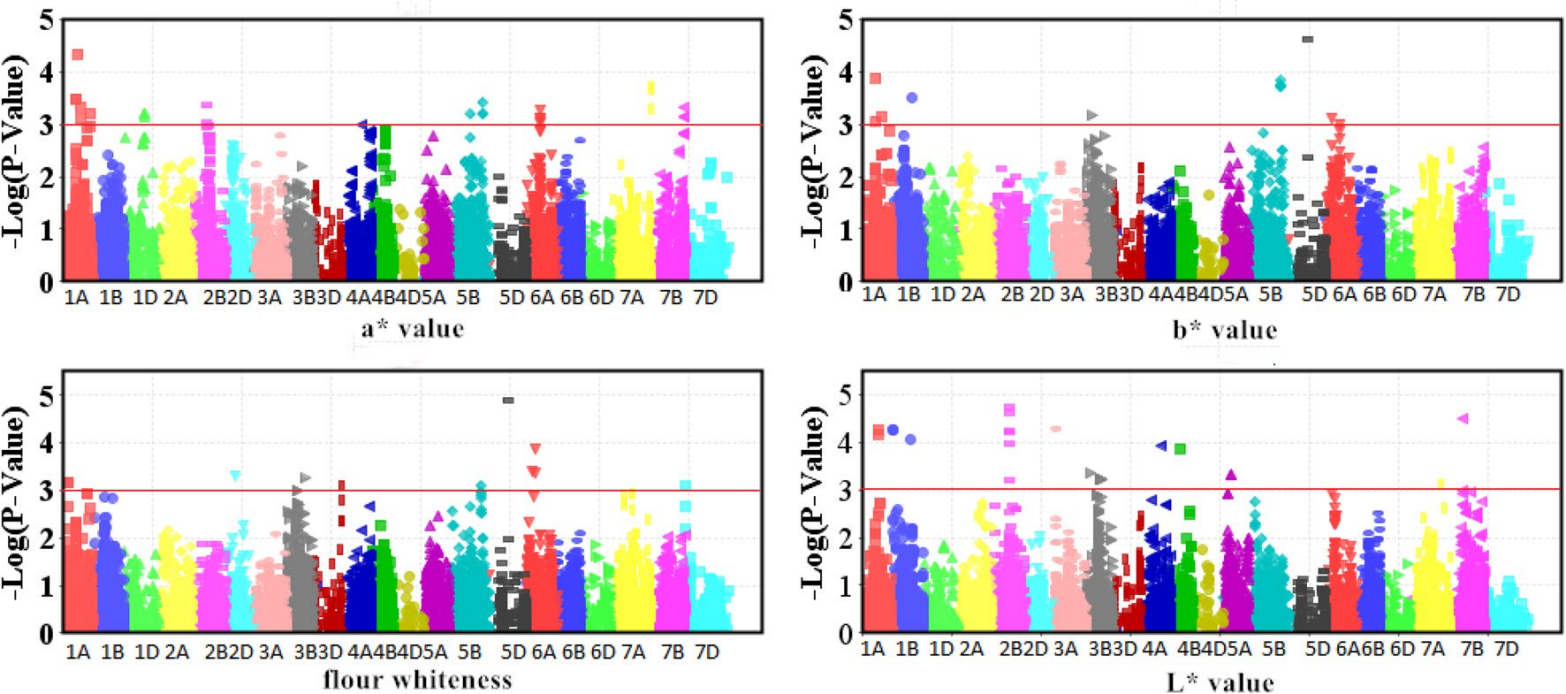
Manhattan plot of flour whiteness and flour colour.

In total, 24,355 mapped SNPs were used for MTA analysis^28,53^. Twenty-two MTAs associated with flour whiteness (P < 10^–4^) were distributed on chromosomes 1A, 1B, 2B, 3A, 5D, 6A, and 7B (Table 3; Fig. 1). There were four SNP loci (*Excalibur_c4709_576, TA001505-1171, BS00000020_51, RAC875_c34446_396*) detected in two or more environments. The *BS00000020_51* locus had the maximum phenotypic variation explained (PVE) with 15.95%. Alleles C and T of marker *BS00000020_51* on chromosome 5D were associated with the largest phenotypic difference (2.13) (Table S2). The phenotypic value of flour whiteness associated with *BS00000020_51-C* on chromosome 5D was significantly higher than that associated with *BS00000020_51-T* across all four environments, indicating that the contribution of *BS00000020_51-*C locus to flour whiteness was better than that of *BS00000020_51-T* locus (Table S2).

**Table 3:** SNP markers significantly associated with flour whiteness in two locations in 2 years (P < 10^–4^).

Marker	Chr.	Pos.	Env.	P-value	R^2^ (%)	Allele	Percentage (%)
Excalibur_c4709_576	2B	99	TA 2015	7.79E−04	5.72	AA/GG	16.1/83.74
			TA 2016	2.38E−05	9.49		
TA001505-1171	2B	99	TA 2016	1.84E−05	9.76	CC/TT	15.61/83.9
			TA 2015	8.24E−04	5.67		
BS00000020_51	5D	103	TA 2015	6.41E−08	15.95	CC/TT	33.66/64.04
			DZ 2015	1.69E−05	10.04		
RAC875_c34446_396	6A	49	DZ 2015	9.05E−04	5.83	AA/GG	59.02/36.59
			DZ 2016	4.36E−04	6.48		
Tdurum_contig43646_147	1A	65	DZ 2015	3.87E−05	9.04	CC/TT	71.71/27.32
RAC875_c65431_351	1A	65	DZ 2015	4.40E−05	8.83	CC/TT	70.73/28.29
Excalibur_c12215_352	1A	65	DZ 2015	4.79E−05	8.73	AA/GG	28.08/71.92
Kukri_c3150_341	1A	65	DZ 2015	4.79E−05	8.73	CC/TT	28.29/71.22
BS00013227_51	1A	65	DZ 2015	4.79E−05	8.73	AA/CC	28.29/71.57
BS00022870_51	1A	65	DZ 2015	4.79E−05	8.73	AA/GG	71.71/28.43
Kukri_c4900_2435	1A	65	DZ 2015	5.26E−05	9.43	GG/TT	20.49/72.68
Tdurum_contig69753_513	1A	65	DZ 2015	6.64E−05	8.64	CC/CC	71.71/25.85
Ra_c105707_788	1A	81	TA 2016	5.47E−05	8.61	CC/TT	18.54/81.46
BS00089894_51	1A	81	TA 2016	6.63E−05	8.41	CC/TT	80.98/19.21
BS00093078_51	1B	8	TA 2016	5.44E−05	8.62	TT/CC	93.66/5.91
Excalibur_c10657_1280	1B	8	TA 2016	5.44E−05	8.62	CC/TT	93.66/5.91
Excalibur_c10657_796	1B	8	TA 2016	5.44E−05	8.62	GG/TT	6.34/93.66
wsnp_BF200640B_Ta_2_1	1B	104	TA 2016	8.69E−05	8.14	AA/GG	6.34/92.68
RAC875_s109189_188	2B	100	TA 2016	5.77E−05	8.56	CC/TT	16.59/82.44
wsnp_RFL_Contig3802_4108582	2B	100	TA 2016	5.96E−05	8.52	CC/TT	16.59/82.93
TA003589-0518	3A	21	TA 2016	5.12E−05	8.68	AA/GG	93.66/5.85
Tdurum_contig10932_913	7B	58	TA 2016	3.14E−05	9.22	AA/CC	63.9/35.12

*Chr.* chromosome, *Pos.* position, *Env.* environment.

In total, 6 SNP loci on chromosomes 1B, 2D, 3A, 5B, 5D and 6A were associated with flour colour brightness (L* value) (P < 10^–4^) (Table 4; Fig. 1). Three SNP loci (*Kukri_c33486_128, BS00000020_51, and GENE-4011_91*) were identified in more than one environment. The *BS00000020_51* locus located on the 5D chromosome was detected in the four environments, with a maximum PVE of 12.28%. Alleles C and T of marker *BS00000020_51* were associated with the largest phenotypic differences (0.57) (Table S2).

**Table 4:** SNP markers significantly associated with the brightness index (L* value) of flour colour in two locations in 2 years (P < 10^–4^).

Marker	Chr.	Pos.	Env.	P-value	R^2^ (%)	Allele	Percentage (%)
Kukri_c33486_128	2D	51	DZ 2015	6.61E−04	6.03	AA/GG	30.73/67.8
			DZ 2016	7.09E−04	6.00		
BS00000020_51	5D	103	TA 2016	2.71E−04	7.32	CC/TT	33.66/64.04
			TA 2015	1.85E−06	12.28		
			DZ 2015	2.18E−04	7.36		
			DZ 2016	4.21E−04	6.57		
GENE-4011_91	6A	49	DZ 2016	5.23E−04	6.69	CC/TT	53.66/41.38
			DZ 2015	1.64E−04	7.65		
Jagger_c1888_277	1B	10	TA 2016	5.66E−05	8.95	CC/TT	21.46/77.34
BS00072153_51	3A	88	DZ 2015	4.78E−05	8.74	AA/GG	27.45/71.43
BS00029348_51	5B	151	TA 2015	3.94E-05	9.23	CC/TT	25.37/69.95

*Chr.* chromosome, *Pos.* position, *Env.* environment.

Seventeen MTAs associated with flour colour redness (a* value) (P < 10^–4^) were distributed on chromosomes 1A, 3A, 6A, 6B, 7A, 7B and 7D. Among them, eleven MTAs were identified in two or more environments (Table 5; Fig. 1). Six SNP loci (*Excalibur_c8883_214, Excalibur_rep_c114255_439, Kukri_c65663_642, Excalibur_rep_c92684_578, Excalibur_c5938_1669, Excalibur_c5938_1703*) were detected in three environments. Alleles A and G of marker *Kukri_c65663_642* were associated with the largest phenotypic differences (0.38) (Table S2).

**Table 5:** SNP markers significantly associated with the redness index (a* value) of flour colour in two locations in 2 years (P < 10^–4^).

Marker	Chr.	Pos.	Env.	P-value	R^2^ (%)	Allele	Percentage (%)
Excalibur_c4152_1031	6A	79	TA 2015	7.67E−05	8.27	TT/CC	11.22/88.78
			DZ 2016	7.55E−04	5.56		
wsnp_Ku_c38451_47086066	6A	79	TA 2015	7.67E−05	8.27	GG/AA	88.78/11.22
			DZ 2016	7.55E−04	5.56		
Excalibur_c8883_214	7A	228	TA 2016	7.12E−06	10.81	AA/GG	87.32/12.32
			DZ 2016	5.10E−04	5.93		
			DZ 2015	1.05E−04	7.57		
Excalibur_rep_c114255_439	7A	228	TA 2016	4.15E−06	11.40	AA/GG	12.2/87.68
			DZ 2016	2.12E−04	6.79		
			DZ 2015	9.10E−05	7.71		
Kukri_c65663_642	7A	233	TA 2016	3.78E−06	11.92	GG/AA	87.32/12.20
			DZ 2016	1.88E−04	6.89		
			DZ 2015	9.27E−05	7.82		
RAC875_rep_c104674_867	7B	171	TA 2016	3.99E−05	8.97	CC/TT	13.66/86.34
			DZ 2015	6.71E−04	5.76		
Excalibur_rep_c92684_578	7B	171	TA 2016	7.83E−06	10.77	AA/GG	84.88/12.86
			DZ 2016	4.76E−04	6.08		
			DZ 2015	1.34E−04	7.50		
BobWhite_c10975_60	7B	171	TA 2016	3.81E−05	9.04	CC/TT	84.88/13.73
			DZ 2015	5.15E−04	6.07		
Excalibur_c5938_1669	7B	171	TA 2016	2.64E−05	9.41	CC/TT	86.34/12.81
			DZ 2016	7.16E−04	5.63		
			DZ 2015	6.30E−04	5.82		
Excalibur_c5938_1703	7B	171	TA 2016	2.64E−05	9.41	GG/TT	13.17/86.34
			DZ 2016	7.16E−04	5.63		
			DZ 2015	6.30E−04	5.82		
Excalibur_c5938_371	7B	171	TA 2016	3.99E−05	8.97	AA/GG	13.66/86.34
			DZ 2015	6.71E−04	5.76		
Ex_c16529_304	1A	79	DZ 2016	4.54E−05	8.66	CC/TT	10.24/84.24
BS00072153_51	3A	88	DZ 2015	6.80E−05	8.03	AA/GG	27.45/71.43
BS00064548_51	6A	83	TA 2015	2.08E−05	9.71	CC/TT	15.2/84.24
Excalibur_c96915_247	6B	119	TA 2015	3.26E−07	15.46	GG/AA	80.49/4.43
BS00027770_51	6B	98	TA 2015	3.78E−05	9.00	GG/AA	5.37/94.63
D_F5XZDLF02FKJFM_220	7D	198	DZ 2015	4.25E−05	8.55	GG/TT	19.51/78.82

*Chr.* chromosome, *Pos.* position, *Env.* environment.

Eleven MTAs on different chromosomes, i.e., 1A, 3B, 4A, 5B, 5D and 6A, were associated with flour colour yellowness (b* value) (P < 10^–4^) (Table 6; Fig. 1). Of these, *TA003858-0637* (6A) and *BS00000020_51* (5D) were found in three and four environments, respectively. Alleles A and G of marker *Kukri_c1214_437* on chromosome 5B were associated with the largest phenotypic differences (0.79). The phenotypic value of flour colour yellowness (b* value) associated with *Kukri_c1214_437-G* was significantly higher than that associated with *Kukri_c1214_437-A* across all four environments, indicating that *Kukri_c1214_437-G* was better than *Kukri_c1214_437-A* for the b* value (Table S2).

**Table 6:** SNP markers significantly associated with the yellowness index (b* value) of flour colour in two locations in 2 years (P < 10^–4^).

Marker	Chr.	Pos.	Env.	P-value	R^2^ (%)	Allele	Percentage (%)
Kukri_c57770_705	1A	103	TA 2015	3.55E−04	6.40	AA/GG	64.88/33.17
			DZ 2016	7.02E−04	5.72		
BS00059383_51	3B	32	DZ 2016	6.84E−04	5.72	CC/AA	66.34/33.33
			DZ 2015	6.95E−04	5.61		
RAC875_c5834_235	4A	151	TA 2015	9.48E−04	5.39	AA/GG	90.24/8.29
			TA 2016	6.84E−04	5.96		
Kukri_c1214_2686	5B	171	TA 2015	3.23E−04	6.38	AA/GG	31.22/68.47
			DZ 2016	1.76E−04	0.07		
Kukri_c1214_437	5B	171	TA 2015	1.98E−04	0.06	AA/GG	30.73/68.97
			DZ 2016	1.47E−04	7.19		
Kukri_c1214_544	5B	171	TA 2015	3.32E−04	6.35	AA/CC	68.78/31.03
			DZ 2016	1.83E−04	6.96		
Kukri_c5228_1011	5B	171	TA 2015	3.66E−04	6.26	CC/TT	31.22/67.32
			DZ 2016	1.94E−04	6.91		
BS00000020_51	5D	103	TA 2015	5.29E−05	8.45	CC/TT	33.66/64.04
			TA 2016	3.19E−04	6.97		
			DZ 2016	2.34E−05	9.10		
			DZ 2015	1.78E−04	6.99		
TA003858-0637	6A	79	TA 2015	1.28E−04	7.49	AA/GG	50.73/48.29
			TA 2016	1.53E−04	7.68		
			DZ 2016	9.83E−04	5.43		
TA005366-0788	6A	79	TA 2015	6.94E−04	5.66	CC/TT	83.9/16.1
			TA 2016	8.96E−04	5.68		
TA005690-1190	6A	79	TA 2015	7.06E−04	5.64	AA/GG	48.78/51.22
			TA 2016	7.35E−04	5.88		

*Chr.* chromosome, *Pos.* position, *Env.* environment.

In general, MTAs consistently identified in more than one environment were considered to be stable. There were four, three, eleven and eleven stable SNP loci for flour whiteness and the L*, a* and b* values, respectively. There was one SNP marker, *BS00000020_51,* associated with both flour whiteness and flour L* and b* values.

### MTAs of the colour traits of dough sheet

**Figure 2:**
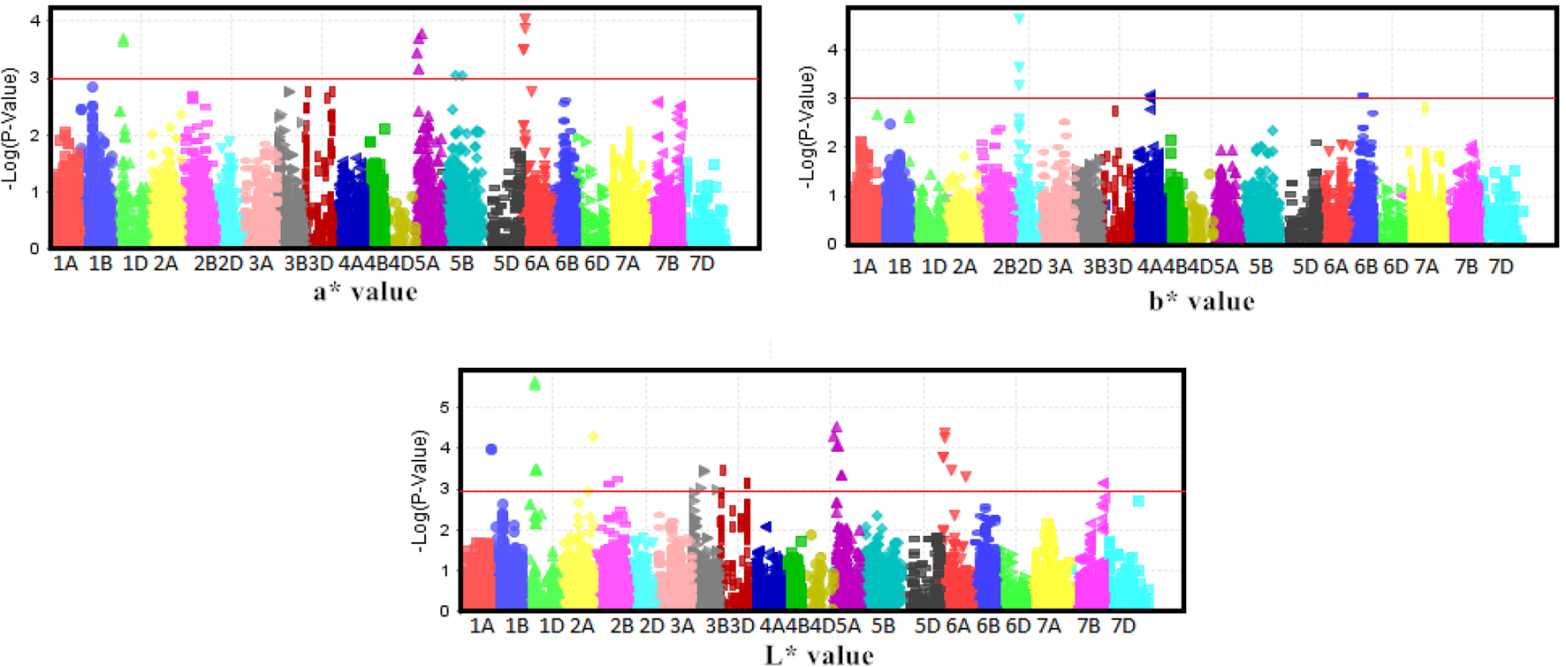
Manhattan plot of the L* value and the a* value of fresh dough sheet and the b* value of dry dough sheet.

Ten MTAs significant at the P ≤ 10^–5^ level for the L* value of dough sheet colour were detected, similar to the a* and b* values of the FDS and DDS (Table 7; Fig. 2). Of these, only one locus, *GENE-1258_171* on chromosome 2A, was found in two environments, with a maximum PVE of 10.51%.

**Table 7:** Loci significantly associated with dough sheet colour in two locations in 2 years.

Trait	Marker	Chr.	Pos.	P value (× 10^–5^)		R^2^ (%)	
				FDS	DDS	FDS	DDS
L* value	BS00099534_51	5A	17	3.06 (E1)	3.05 (E1)	9.00 (E1)	9.00 (E1)
	BobWhite_c15802_72	6A	27	5.38 (E1)	5.58 (E1)	8.43 (E1)	8.39 (E1)
	BobWhite_c23992_300	5A	17	9.01 (E1)	9.04 (E1)	7.89 (E1)	7.89 (E1)
	BobWhite_rep_c67379_241	5A	7	4.89 (E1)	4.87 (E1)	8.53 (E1)	8.54 (E1)
	BS00065510_51	1D	68	0.23 (E1)	0.24 (E1)	12.46 (E1)	12.42 (E1)
	BS00065722_51	1D	68	0.28 (E1)	0.28 (E1)	11.56 (E1)	11.53 (E1)
	GENE-1258_171	2A	184	5.20 (E1)/4.66 (E3)	5.06 (E1)/4.35 (E3)	10.48 (E1)/9.25 (E3)	10.51 (E1)/9.36 (E3)
	Kukri_c102502_261	6A	27	4.20 (E1)	4.38 (E1)	9.20 (E1)	9.16 (E1)
	Kukri_c16477_181	2D	40	–	2.24 (E2)	–	9.31 (E2)
	RAC875_c106584_1077	5A	32	8.95 (E1)	9.32 (E1)	7.90 (E1)	7.86 (E1)
a* value	RAC875_c106584_1077	5A	32	16.64 (E1)	16.68 (E1)	7.13 (E1)	7.13 (E1)
	Kukri_c16477_181	2D	40	–	2.31 (E2)	–	9.28 (E2)
	Kukri_c102502_261	6A	27	9.30 (E1)	9.30 (E1)	8.36 (E1)	8.35 (E1)
	GENE-1258_171	2A	184	48.57 (E3)	46.89 (E3)	9.39 (E3)	9.25 (E3)
	BS00099534_51	5A	17	20.52 (E1)	20.47 (E1)	6.93 (E1)	6.93 (E1)
	BS00065722_51	1D	68	22.74 (E1)	22.71 (E1)	6.86 (E1)	6.87 (E1)
	BS00065510_51	1D	68	20.48 (E1)	20.46 (E1)	7.26 (E1)	7.26 (E1)
	BobWhite_rep_c67379_241	5A	7	35.64 (E1)	35.57 (E1)	6.40 (E1)	6.41 (E1)
	BobWhite_c23992_300	5A	17	67.42 (E1)	67.36 (E1)	5.78 (E1)	5.78 (E1)
	BobWhite_c15802_72	6A	27	13.21 (E1)	13.21 (E1)	7.37 (E1)	7.37 (E1)
b* value	BS00099534_51	5A	17	20.95 (E1)	20.76 (E1)	6.91 (E1)	6.92 (E1)
	BobWhite_c15802_72	6A	27	13.19 (E1)	7.37 (E1)	12.87 (E1)	7.40 (E1)
	BobWhite_c23992_300	5A	17	69.00 (E1)	67.85 (E1)	5.75 (E1)	5.77 (E1)
	BobWhite_rep_c67379_241	5A	7	36.4 (E1)	36.13 (E1)	6.39 (E1)	6.39 (E1)
	BS00065510_51	1D	68	20.73 (E1)	20.67 (E1)	7.25 (E1)	7.25 (E1)
	BS00065722_51	1D	68	23.04 (E1)	22.99 (E1)	6.85 (E1)	6.85 (E1)
	GENE-1258_171	2A	184	47.18 (E3)	47.78 (E3)	9.24 (E3)	9.22 (E3)
	Kukri_c102502_261	6A	27	9.27 (E1)	9.03 (E1)	8.36 (E1)	8.39 (E1)
	Kukri_c16477_181	2D	40	–	2.34 (E2)	–	9.27 (E2)
	RAC875_c106584_1077	5A	32	16.79 (E1)	16.32 (E1)	7.12 (E1)	7.15 (E1)

E1, TA 2015; E2, DZ 2015; E3, TA 2016; E4, DZ 2016. *Chr*. chromosome, *Pos.* position, *Env.* Environment, L* value the brightness index, a* value the redness index, b* value the yellowness index, *FDS* fresh dough sheet, *DDS* dry dough sheet.

### Prediction of candidate genes for colour traits of flour

Markers with high PVE values were selected from loci significantly associated with flour whiteness and flour colour for prediction (Table S3). Some important loci were identified. There were twenty-one candidate genes predicated. Of which, the marker *BS00000020_51* on chromosome 5D had two candidate genes, *TraesCS5D01G004300* and *Gsp-1D*, from wheat (Table S3). The functions of these genes are related to grain hardness, that is, puroindoline-b and an arabinogalactan peptide, respectively. The candidate gene of the marker *Excalibur_c4709_576* on 2B in wheat and *Arabidopsis* (mouse-ear cress) also participates in glycerol-3-phosphate O-acyltransferase activity and protein self-association. The marker *BS00027770_51* on chromosome 6B had the candidate gene *TraesCS6B01G419200*, whose functions is related to the flavonoid biosynthetic process and regulation of jasmonic acid mediated signalling pathway. The candidate gene *TraesCS5A01G003800* for the marker *BS00099534_51* on chromosome 5A is related to the fucose metabolic process and protein phosphorylation. The marker *TraesCS6A01G241200* on chromosome 6A had the candidate gene *TA005690-1190*, whose function is related to the ketone body biosynthetic process, leucine catabolic process and lipid metabolic process. These candidate genes may be related to flour colour, and their functions will be explored in future research.

### Prediction of candidate genes for dough sheet colour

Markers with high PVE values were selected from loci significantly associated with dough sheet colour for prediction. Five new genes have been predicated. The marker *BS00065510_51* on chromosome 1D had the candidate gene *TraesCS1D01G070200* from wheat (Table S4). The functions of this gene are related to ATP binding, RNA binding and RNA helicase activity. The candidate gene *TraesCS1D01G070300* of the marker *BS00065722_51* on 1D in *Oryza barthii* also participates in the RNA catabolic process. The marker *GENE-1258_171* on chromosome 2A had the candidate gene *TraesCS2A01G593500* from *Oryza barthii*, *Aegilops tauschii* and wheat. The functions of this gene are related to protein tyrosine kinase activity and protein serine/threonine kinase activity. The gene TraesCS5A01G003800 is predicated on chromosome 5A for maker BS00099534_51, whose function is related to catalytic activity and transferase activity participating in fucose metabolic process. The marker *BobWhite_c15802_72* on chromosome 6A had the candidate gene *TraesCS6A01G024900* from wheat, soybean (*Glycine hispida*) and Arabidopsis (mouse-ear cress). The functions of this gene are related to the response to oxidative stress, adenine salvage activity and the carbohydrate metabolic process. These candidate genes may be related to dough sheet colour, and their functions will be explored in future research.

## Discussion

Flour whiteness and flour and dough colour-related traits are critical determinants for the end-use product quality of wheat. Therefore, it is important to identify some major and stable loci for these traits and then transfer these favourable alleles into commercial varieties. Although some loci for these traits were found in previous studies in different populations^11,30–34^ using QTL mapping methods, genome-wide association studies (GWAS) for these traits are rarely conducted using single nucleotide polymorphism markers (SNPs). Therefore, it is still important to find new major and stable loci introgressed into commercial cultivars using GWAS.

Zhai et al.^34^ constructed a genetic map that included 8227 SNP markers using an RIL population and found fifty-six QTLs. However, in the present study, a total of 24,355 SNP markers were mapped for MTA analysis using a panel of varieties, and more new loci were found than in a previous study. Some chromosomes for flour colour traits were involved in QTL mapping, including mainly homoeologous group 1, 2, 5, and 7 chromosomes, and chromosomes 3B, 4A, 4B and 6B^34,35^. Some QTLs detected for the a* value were found on the 1B and 3B chromosomes^36^, and other involved chromosomes were mainly 1B, 1D, 2D, 4A, 4D and 7B. In this study, loci associated with these traits were found on chromosomes 6A, 4A and 3A in addition to the above chromosomes. Zhai et al.^37^ performed GWAS on 166 bread wheat cultivars using the wheat 90 and 660K SNP arrays and 10 allele-specific markers, and identified 100 MTAs for flour color-related traits. This indicated that GWAS and QTL mapping could be complementary to each other.

PPO activity and the yellow pigment content have been reported to affect flour whiteness and colour^38–40^. Previous studies showed that PPO activity is mainly controlled by the genes on the homoeologous group 2 chromosomes, particularly 2A and 2D^41,42^. In the present study, significant SNP loci markers on chromosomes 2B, 2D and 2A were associated with flour whiteness, the flour L* value and dough sheet colour, but these SNP loci showed be different from PPO genes loci by comparing their physical positions. *Excalibur_c4709_576* and *TA001505-1171* on chromosome 2B were significantly associated with flour whiteness in two environments. Their candidate gene prediction indicated that the function was related to glycerol-3-phosphate O-acyltransferase activity, which participates in the fatty acid biosynthetic process. Previous research showed that lipoxygenase affects flour whiteness and colour^43^. Therefore, these loci may influence flour whiteness by lipoxygenase but not PPO activity. *Kukri_c33486_128,* on chromosome 2D, which was significantly associated with the flour L* value, was stably identified in three environments, but its candidate gene and function were not predicted in the BLAST search, which indicated that this locus is new and should be further studied in the future. *GENE-1258_171* on chromosome 2A was significantly associated with the dough sheet L* value in two environments. Its predicted gene is *TraesCS2A01G593500*, whose function is related to protein kinase activity, so this locus affects the dough sheet colour, perhaps as a result of the grain protein content.

Previous studies showed that the yellow pigment content is mainly controlled by chromosomes 7A and 7B; moreover, the Psy1 gene was reported to co-segregate with the b* value and yellow pigment content^35,44,45^ In the present study, significantly associated loci were also found on these two chromosomes. Only one SNP marker associated with flour whiteness was detected on chromosome 7B. Although no loci were found on these two chromosomes for the flour b* value, nine SNP markers were significantly associated with the flour a* value. Moreover, they seemed to be stable in multiple environments. Three SNP markers were found on chromosome 7A, and their function is related to leucine rich repeat family protein expression. The other six SNP markers on chromosome 7B have one candidate gene, *TraesCS7B01G482200*, which is related to sucrose synthase activity. Therefore, the mechanism of their influence on flour colour is different from that of the Psy gene and yellow pigment content, which needs to be further studied in the future.

In addition, the flour whiteness colour is also affected by milling characteristics^32,46^. However, the milling characteristics are influenced by grain hardness, so the grain hardness affects the flour whiteness and colour. Zhai et al. identified the QTL *QFL.caas-5D-1*, which is close to the Pin-b gene, with a distance of 2.1 cM. The QTL for the L* value on chromosome 5DS coincided with the hardness (Ha) locus in previous studies^32,46^. In the present study, the SNP marker *BS00000020_51* on chromosome 5D was significantly associated with flour whiteness and the L* and b* values and was stably detected in multiple environments. Therefore, this locus is important for flour whiteness and colour. Through a comparison of Zhai et al.’s results^34^, this SNP marker (*BS00000020_51*) was also found on their genetic map and was close to the closest marker *BobWhite_s67669_117* of the QTL, with a distance of 2.1 cM. This indicated that the SNP marker *BS00000020_*51 controls flour whiteness and colour through grain hardness. We found two candidate genes, *TraesCS5D01G004300* and *Gsp-1D*, by candidate gene prediction. Of these, *Gsp-1D* is the grain softness protein-1 gene that is linked to grain hardness, which affects flour particle size. Moreover, flour whiteness showed negatively correlated with flour particle size^47^, so it is possible that *Gsp-1D* affect the flour whiteness. Therefore, the accuracy of the results is confirmed.

Most interestingly, the SNP markers found on chromosome 6A were significantly associated with flour whiteness and colour. The markers *RAC875_c34446_396* and *GENE-4011_91* were at the same position, i.e., 49 cM, and were associated with flour whiteness and the flour L* value, respectively. The function of the candidate gene *PUP88* was related to hydrolase activity and the hydrolysis of O-glycosyl compounds, participating in the carbohydrate metabolic process. However, at position 79 cM, five SNP markers detected in more than one environment were associated with the flour a* and b* values. The functions of the candidate gene *TraesCS6A01G241200* of the SNP marker *TA005690-1190* were different from those of other candidate genes. The *TraesCS6A01G241200* gene participates in the ketone body biosynthetic process and lipid metabolic processes, which affect flour colour. Other candidate genes were mainly related to proteins. Moreover, the SNP marker *BobWhite_c15802_72* on chromosome 6A was associated with dough sheet colour. Its function is related to peroxidase activity in soybean, but in wheat, the biological process is not clear. In addition, one special locus, the *BS00027770_51* marker, associated with the flour a* value, was identified on chromosome 6B. Its candidate gene participates in the flavonoid biosynthetic process and regulation of jasmonic acid in *Arabidopsis thaliana*, but the function in wheat remains unknown. The above loci were not found in previous studies.

Flour whiteness and colour-related traits are inherently correlated. Three important loci on chromosomes 5D and 6A were identified. The SNP locus at genetic position 103 cM of chromosome 5D was involved in both flour whiteness and the b* value; the loci at genetic position 49 cM of chromosome 6A influenced flour whiteness and the flour L* value, and the last locus at genetic position 79 cM of chromosome 6A was associated with the flour a* and b* values. These relationships were reflected by the correlation coefficients (Table S5; Table S6), in agreement with previous studies^34^. Their physical positions were seen in Table S7. Therefore, genes with pleiotropic effects may explain the genetic basis of trait correlation. Pleiotropic effects were observed for dough sheet colour-related traits.

## Conclusions

This study provided the important information about the influence of proteins, lipoxygenase and grain hardness on the flour whiteness and colour in addition to PPO activity and yellow pigment at the molecular level. GWAS is a good method for identifying new, important, stable loci. SNP markers significantly associated with flour whiteness and colour detected in this study provide opportunities for MAS of traits that are difficult to phenotype at the early stages of wheat breeding.

## Materials and methods

### Ethics statement

All samples analysed in our study adhered to all local, national or international guidelines and legislation, and no ethical approval was required.

### Plant material

The association mapping panel of 205 wheat genotypes for GWAS comprised 77 released cultivars, 55 landraces including two lines from Mexico and France, and 73 breeding lines from 10 provinces that represent the major winter wheat production regions in China^27,28,48^.

### Growth conditions

The seeds used for the association mapping panel were planted in the 2015 and 2016 growing seasons in the experimental fields of two locations, that is, the Shandong Agricultural University, Tai’an (TA) location (116°36′ E, 36°57′ N) and the Dezhou Institute of Agricultural Sciences, Dezhou (DZ) location (116°29′ E, 37°45′ N). E1, E2, E3 and E4 represented Tai’an location in 2015 (TA 2015), Dezhou loction in 2015 (DZ 2015), Tai’an location in 2016 (TA 2016), and Dezhou location in 2016 (DZ 2016), respectively. The experimental field was arranged in a completely randomized block design, with two replicates in each environment. All lines were grown in 2 m plots with 3 rows spaced 25 cm apart, and 70 seeds were evenly broadcast in each row. During the growing seasons, all recommended local crop management practices were followed, and damage attributed to lodging, disease, or pests was not observed.

### Phenotypic trait evaluation

Flour milling was carried out by using a Bühler experimental mill (Bühler mill, Bühler-Miag Company, Braunschweig, Germany) with a flour extraction yield of approximately 70% in all samples stored for approximately 1 month after being harvested. The samples were tempered to 14%-16% moisture content according to grain texture overnight before milling.

Flour whiteness was determined by a WSB-IV intelligent whiteness determination meter (Dajiguangdian Instruments, Hangzhou, China)^36^. The working principle is to measure the absolute spectral diffuse reflectance using a photometry integrating sphere. The peak wavelength of the spectral power distribution of Y10 whiteness optical system is 475 nm and the half wave width is 44 nm. The standard of Y10 optical system accords with national standard GB3979 of China.

Dough making was performed according to^49^ with minor modifications. Flour and water were mixed to achieve 44% absorption by slow mixing at a low speed for 5 min, followed by mixing at a medium speed for 2 min using a Kitchen Aid Professional Mixer (KPM5, St. Joseph, MI, USA). During the resting stage, crumbly dough was placed in a stainless steel bowl for 20 min at room temperature. The crumbly dough was then hand kneaded into a stiff mass and passed through an automatic noodle maker (JMTZ-14, Dongfang Fude Technology Development Center, Beijing, China) three times to form a noodle sheet at a 2.0 mm roll gap-setting. The dough sheet was then folded twice and passed through six different roll gaps (3.5, 3.0, 2.5, 2.0, 1.5, and 1.0 mm). Then, the fresh dough sheet was cut into approximately 6 small sheets (length 10 cm, width 5 cm, thickness 1.0 mm). The fresh sheet was dried in the oven for 24 h at 40 °C.

### The colour-related traits of the flour and dough sheet

The flour colour parameters (L*, a* and b*) were measured with a Minolta colorimeter (CR-300, Minolta Camera Co., Ltd., Osaka, Japan) using the commission Internationale de l’éclairage (CIE) L* a* b* colour system^50^. The L* value indicates the lightness of flour with a range of 0–100 representing darkness to lightness (L* = 0 means black, L* = 100 means white, and the middle value is a grey transition with different brightness). The a* value indicates the red-green direction, that is, it designates redness when positive but greenness when negative. The b* value indicates the degree of the flour yellow-blue colour, that is, the higher b* value denotes a greater amount of yellow^51^.

The dough sheet colour was also measured by a Minolta CR-300 colour meter. Three points were measured to determine the fresh dough sheet (FDS) (uncooked) at 0 h and the dry dough sheet colour at 24 h per noodle sheet, each at a different location on the same side of the surface of the noodle sheet^52^.

The colorimeter parameter of each sample was measured three times, and the mean values were used for subsequent statistical analysis.

### Statistical analysis

Analysis of variance (ANOVA) and correlations among phenotypic traits were carried out using the statistical software SPSS version 17.0 (SPSS Inc., Chicago, IL, USA). Heritability (*h*^2^) was calculated as h_B_^2^ = σ_g_^2^/(σ_g_^2^ + σ_ge_^2^/r + σ_e_^2^/r_e_), where σ_g_^2^, σ_ge_^2^, and σ_e_^2^ are estimates of genotype, genotype × environment and residual error variances, respectively, r is replicates, and re is the product of replicates and number of environments. The estimates of σ_g_^2^, σ_ge_^2^, and σ_e_^2^ were obtained from variance estimates included in the ANOVA, which was performed using the PROC GLM procedure of SAS 8.0 (SAS Institute Inc., Cary, NC, USA).

### Genome-wide association analysis

SNP markers, genotyping and the population structure of the samples were reported previously^27,28^. Based on this information, significant marker–trait associations (MTAs) were identified using a mixed linear model (MLM) in TASSEL3.0. The P-value was used to determine whether a QTL was associated with a marker. The R^2^ value was used to evaluate the magnitude of the MTA effects. The genome-wide significance threshold (P-value ≤ 0.001) was given. SNPs with P-value ≤ 0.001 were considered to be significantly associated with phenotypic traits. When the MTA locus was detected in two or more environments, it was considered a stable association site^48^.

### Forecasting candidate genes for flour whiteness and dough sheet colour-related traits

To identify the position of important MTA loci on a physical map and possible candidate genes, significant markers detected in this study were used to identify putative candidate genes. A BLAST (Basic Local Alignment Search Tool) search was performed on the International Wheat Genome Sequencing Consortium database (IWGSC; http://www.wheatgenome.org/, 20th November 2020) using the sequence of the significant SNP markers identified by GWAS. When an SNP marker sequence from the IWGSC was 100% identical to any wheat contig, the sequence was extended 2 Mb for each marker using the IWGSC BLAST results. Then, the extended sequence was used to run a BLAST search at the National Center for Biotechnology Information (NCBI) database (http://www.ncbi.nlm.nih.gov, 20th November 2020) and Ensembl Plants (http://plants.ensembl.org/Triticum_aestivum/Tools/Blast, 20th November 2020) to confirm possible candidate genes and functions.

### Ethics approval and consent to participate

Wheat is a common crop extensively cultivated in the world. This study does not contain any research requiring ethical consent or approval.

## Supplementary Information

Supplementary Information.

## Data Availability

All data used during the current study are included in this published article or are available from the corresponding author on reasonable request.
