# Supplementary material for: Genome wide association study of the whiteness and colour related traits of flour and dough sheets in common wheat
Source: Sci Rep. 2021 Apr 22;11:8790. doi: 10.1038/s41598-021-88241-4 (PMC8062544; doi:10.1038/s41598-021-88241-4)
Supplement: Supplementary file 1 — Supplementary Information. [file 41598_2021_88241_MOESM1_ESM.docx]

**Supplementary Tables and Figures**


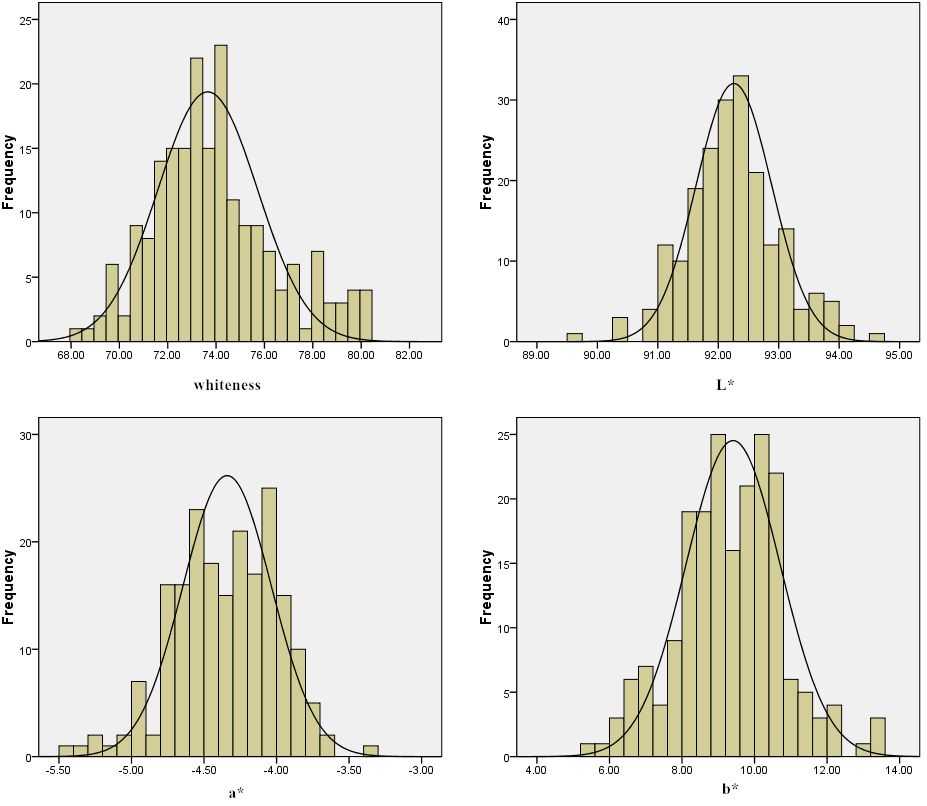


**Fig.S1** The frequency distribution of the whiteness, L*, a*, and b* value of flour


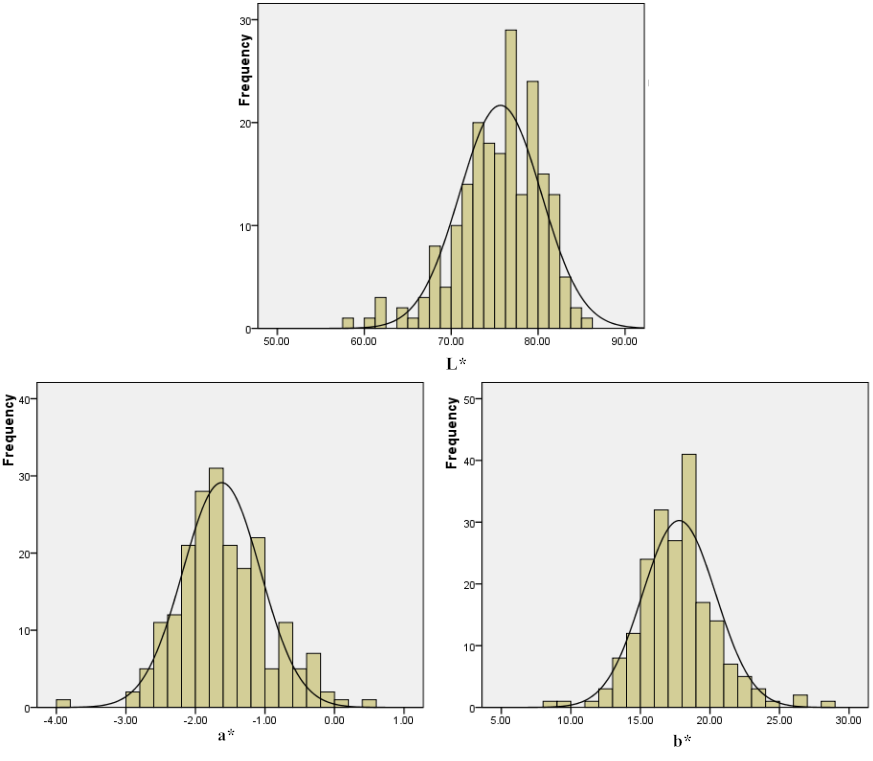


**Fig.S2** The frequency distribution of L*, a* and b* of dough sheet color

**Table S1** variance analysis for colour traits of flour and dough sheet

|  | **Trait Mean squares** | | | | | |  |
| --- | --- | --- | --- | --- | --- | --- | --- |
|  |  | Genotypes  (df=204) | Environment  (df=3) | Replicate  (df=8) | G×E  (df=578) | Error | *h^2^*(%) |
| flour | whiteness | 55.16*** | 609.487*** | 552.53*** | 4.97*** | 0.47*** | 98.5 |
|  | L*value | 198.71*** | 59952.21*** | 234.52*** | 183.78*** | 6.57*** | 24.2 |
|  | a*value | 2.47*** | 7658.10*** | 0.18*** | 2.17 | 0.47*** | - |
|  | b*value | 16.13*** | 947.30*** | 22.03*** | 1.07*** | 0.02*** | 99.1 |
| FDS | L*value | 64.18*** | 539.19*** | 5.28*** | 36.33*** | 0.07*** | 28.9 |
|  | a*value | 1.88*** | 48.63*** | 28.65*** | 0.97*** | 0.24*** | 50.0 |
|  | b*value | 31.41*** | 2605.87*** | 3.61*** | 14.46*** | 0.03*** | 48.1 |
| DDS | L*value | 80.37*** | 3365.92*** | 9.37*** | 67.35*** | 0.11*** | - |
|  | a*value | 1.148*** | 22.63*** | 0.72*** | 1.25*** | 0.1*** | - |
|  | b*value | 50.49*** | 999.42*** | 34.96*** | 23.97*** | 0.39*** | 46.0 |

FDS: Fresh dough sheet, DDS: dry dough sheet. ***, Significant at P < 0.0001

**Table S2** Phenotypic differences of alleles at flour and dough sheet

| Locus | Chr. | Allele | Number of genotypes | whiteness | | | | | | a* Value | | | | | | b* Value | | | | | | L* Value | | | | | |
| --- | --- | --- | --- | --- | --- | --- | --- | --- | --- | --- | --- | --- | --- | --- | --- | --- | --- | --- | --- | --- | --- | --- | --- | --- | --- | --- | --- |
|  |  |  |  | Env. | | | | Average | Difference | Env. | | | | Average | Difference | Env. | | | | Average | Difference | Env. | | | | Average | Difference |
|  |  |  |  | E1 | E2 | E3 | E4 |  |  | E1 | E2 | E3 | E4 |  |  | E1 | E2 | E3 | E4 |  |  | E1 | E2 | E3 | E4 |  |  |
| Excalibur_c4709_576 | 2B | A | 33 | 73.14Aa | 74.86Aa | 74.35Aa | 75.38Aa | 74.43 | 0.44 |  |  |  |  |  |  |  |  |  |  |  |  |  |  |  |  |  |  |
|  |  | G | 172 | 74.08Aa | 74.52Aa | 75.11Aa | 75.75Aa | 74.87 |  |  |  |  |  |  |  |  |  |  |  |  |  |  |  |  |  |  |  |
| TA001505-1171 | 2B | T | 172 | 74.08Aa | 74.91Aa | 75.11Aa | 75.75Aa | 74.96 | 0.58 |  |  |  |  |  |  |  |  |  |  |  |  |  |  |  |  |  |  |
|  |  | C | 32 | 73.19Aa | 74.52Aa | 74.39Aa | 75.40Aa | 74.38 |  |  |  |  |  |  |  |  |  |  |  |  |  |  |  |  |  |  |  |
| BS00000020_51 | 5D | C | 69 | 76.09Aa | 75.64Aa | 76.86Aa | 76.23Aa | 76.21 | 2.13 |  |  |  |  |  |  | 9.92Aa | 10.17Aa | 6.57Aa | 7.10Aa | 8.44 | 0.23 | 92.72Aa | 92.88Aa | 74.10Aa | 74.03Aa | 83.43 | 0.57 |
|  |  | T | 132 | 73.67Bb | 72.98Bb | 75.17Bb | 74.49Bb | 74.08 |  |  |  |  |  |  |  | 8.65Bb | 8.90Bb | 7.39Bb | 7.88Bb | 8.21 |  | 91.87Bb | 92.28Bb | 73.64Bb | 73.66Bb | 82.86 |  |
| RAC875_c34446_396 | 6A | A | 121 | 74.19Aa | 75.08Aa | 75.42Aa | 76.23Aa | 75.23 | 1.03 |  |  |  |  |  |  |  |  |  |  |  |  |  |  |  |  |  |  |
|  |  | G | 75 | 73.65Aa | 73.74Bb | 74.45Bb | 74.97Bb | 74.20 |  |  |  |  |  |  |  |  |  |  |  |  |  |  |  |  |  |  |  |
| Kukri_c33486_128 | 2D | A | 63 |  |  |  |  |  |  |  |  |  |  |  |  |  |  |  |  |  |  | 92.50Aa | 92.76Aa | 73.97Aa | 74.02Aa | 83.31 | 0.38 |
|  |  | G | 140 |  |  |  |  |  |  |  |  |  |  |  |  |  |  |  |  |  |  | 92.12Bb | 92.20Bb | 73.78Ab | 73.61Bb | 82.93 |  |
| GENE-4011_91 | 6A | C | 121 |  |  |  |  |  |  |  |  |  |  |  |  |  |  |  |  |  |  | 92.33Aa | 92.58Aa | 73.85Aa | 73.97Aa | 83.18 | 0.28 |
|  |  | T | 75 |  |  |  |  |  |  |  |  |  |  |  |  |  |  |  |  |  |  | 92.15Aa | 92.10Bb | 73.80Aa | 73.56Bb | 82.90 |  |
| Excalibur_c4152_1031 | 6A | C | 128 |  |  |  |  |  |  | -4.35Aa | -4.47Aa | -0.85Aa | -0.66Aa | -2.58 | 0.26 |  |  |  |  |  |  |  |  |  |  |  |  |
|  |  | T | 23 |  |  |  |  |  |  | -4.39Aa | -3.39Bb | -0.85Aa | -0.66Aa | -2.32 |  |  |  |  |  |  |  |  |  |  |  |  |  |
| wsnp_Ku_c38451_47086066 | 6A | A | 23 |  |  |  |  |  |  | -4.35Aa | -4.47Aa | -0.85Aa | -0.66Aa | -0.35 | 0.28 |  |  |  |  |  |  |  |  |  |  |  |  |
|  |  | G | 182 |  |  |  |  |  |  | -4.39Aa | -3.39Bb | -0.85Aa | -0.66Aa | -0.63 |  |  |  |  |  |  |  |  |  |  |  |  |  |
| Excalibur_c8883_214 | 7A | A | 179 |  |  |  |  |  |  | -4.30Aa | -4.37Aa | -0.81Aa | -0.61Aa | -0.34 | 0.3 |  |  |  |  |  |  |  |  |  |  |  |  |
|  |  | G | 26 |  |  |  |  |  |  | -4.75Bb | -4.20Aa | -1.16Bb | -0.96Bb | -0.67 |  |  |  |  |  |  |  |  |  |  |  |  |  |
| Excalibur_rep_c114255_439 | 7A | A | 25 |  |  |  |  |  |  | -4.76Bb | -4.20Aa | -1.17Bb | -0.98Bb | -0.68 | 0.34 |  |  |  |  |  |  |  |  |  |  |  |  |
|  |  | G | 179 |  |  |  |  |  |  | -4.30Aa | -4.37Aa | -0.81Aa | -0.61Aa | -0.34 |  |  |  |  |  |  |  |  |  |  |  |  |  |
| Kukri_c65663_642 | 7A | A | 25 |  |  |  |  |  |  | -4.77Bb | -4.20Aa | -1.17Bb | -0.98Bb | -0.68 | 0.38 |  |  |  |  |  |  |  |  |  |  |  |  |
|  |  | G | 179 |  |  |  |  |  |  | -4.30Aa | -4.37Aa | -0.81Aa | -0.61Aa | -0.33 |  |  |  |  |  |  |  |  |  |  |  |  |  |
| RAC875_rep_c104674_867 | 7B | C | 28 |  |  |  |  |  |  | -4.68Bb | -4.20Aa | -1.17Bb | -0.98Bb | -0.66 | 0.32 |  |  |  |  |  |  |  |  |  |  |  |  |
|  |  | T | 177 |  |  |  |  |  |  | -4.30Aa | -4.37Aa | -0.81Aa | -0.61Aa | -0.34 |  |  |  |  |  |  |  |  |  |  |  |  |  |
| Excalibur_rep_c92684_578 | 7B | A | 174 |  |  |  |  |  |  | -4.29Aa | -4.41Aa | -0.81Aa | -0.61Aa | -0.33 | 0.33 |  |  |  |  |  |  |  |  |  |  |  |  |
|  |  | G | 26 |  |  |  |  |  |  | -4.75Bb | -4.22Aa | -1.16Bb | -0.96Bb | -0.66 |  |  |  |  |  |  |  |  |  |  |  |  |  |
| BobWhite_c10975_60 | 7B | C | 174 |  |  |  |  |  |  | -4.30Aa | -4.41Aa | -0.81Aa | -0.61Aa | -0.33 | 0.32 |  |  |  |  |  |  |  |  |  |  |  |  |
|  |  | T | 28 |  |  |  |  |  |  | -4.68Bb | -4.22Aa | -1.16Bb | -0.96Bb | -0.65 |  |  |  |  |  |  |  |  |  |  |  |  |  |
| Excalibur_c5938_1669 | 7B | C | 177 |  |  |  |  |  |  | -4.30Aa | -4.41Aa | -0.81Aa | -0.61Aa | -0.33 | 0.32 |  |  |  |  |  |  |  |  |  |  |  |  |
|  |  | T | 27 |  |  |  |  |  |  | -4.70Bb | -4.23Aa | -1.16Bb | -0.96Bb | -0.65 |  |  |  |  |  |  |  |  |  |  |  |  |  |
| Excalibur_c5938_1703 | 7B | G | 177 |  |  |  |  |  |  | -4.70Bb | -4.21Aa | -1.12Bb | -0.94Bb | -0.64 | 0.3 |  |  |  |  |  |  |  |  |  |  |  |  |
|  |  | T | 27 |  |  |  |  |  |  | -4.30Aa | -4.37Aa | -0.81Aa | -0.62Aa | -0.34 |  |  |  |  |  |  |  |  |  |  |  |  |  |
| Excalibur_c5938_371 | 7B | A | 28 |  |  |  |  |  |  | -4.68Bb | -4.23Aa | -1.12Bb | -0.92Bb | -0.62 | 0.28 |  |  |  |  |  |  |  |  |  |  |  |  |
|  |  | G | 177 |  |  |  |  |  |  | -4.30Aa | -4.37Aa | -0.81Aa | -0.62Aa | -0.34 |  |  |  |  |  |  |  |  |  |  |  |  |  |
| Kukri_c57770_705 | 1A | A | 133 |  |  |  |  |  |  |  |  |  |  |  |  | 9.77Aa | 9.43Aa | 7.69Aa | 7.15Aa | 8.51 | 0.32 |  |  |  |  |  |  |
|  |  | G | 68 |  |  |  |  |  |  |  |  |  |  |  |  | 9.30Ab | 9.09Aa | 7.42Aa | 6.94Aa | 8.19 |  |  |  |  |  |  |  |
| BS00059383_51 | 3B | A | 68 |  |  |  |  |  |  |  |  |  |  |  |  | 9.05Bb | 8.75Bb | 7.17Bb | 6.69Bb | 7.92 | 0.74 |  |  |  |  |  |  |
|  |  | C | 136 |  |  |  |  |  |  |  |  |  |  |  |  | 9.94Aa | 9.62Aa | 7.82Aa | 7.27Aa | 8.66 |  |  |  |  |  |  |  |
| Kukri_c1214_2686 | 5B | A | 64 |  |  |  |  |  |  |  |  |  |  |  |  | 9.13Bb | 8.91Bb | 7.33Ab | 6.75Bb | 8.03 | 0.55 |  |  |  |  |  |  |
|  |  | G | 140 |  |  |  |  |  |  |  |  |  |  |  |  | 9.86Aa | 9.52Aa | 7.72Aa | 7.23Aa | 8.58 |  |  |  |  |  |  |  |
| Kukri_c1214_437 | 5B | A | 63 |  |  |  |  |  |  |  |  |  |  |  |  | 9.09Bb | 8.87Bb | 7.29Bb | 6.73Bb | 7.80 | 0.79 |  |  |  |  |  |  |
|  |  | G | 141 |  |  |  |  |  |  |  |  |  |  |  |  | 9.87Aa | 9.52Aa | 7.72Aa | 7.23Aa | 8.59 |  |  |  |  |  |  |  |
| Kukri_c1214_544 | 5B | A | 141 |  |  |  |  |  |  |  |  |  |  |  |  | 9.87Aa | 9.52Aa | 7.72Aa | 7.23Aa | 8.59 | 0.56 |  |  |  |  |  |  |
|  |  | C | 64 |  |  |  |  |  |  |  |  |  |  |  |  | 9.13Bb | 8.91Bb | 7.33Ab | 6.75Bb | 8.03 |  |  |  |  |  |  |  |
| Kukri_c5228_1011 | 5B | C | 65 |  |  |  |  |  |  |  |  |  |  |  |  | 9.14Bb | 8.92Bb | 7.34Ab | 6.76Bb | 8.04 | 0.54 |  |  |  |  |  |  |
|  |  | T | 140 |  |  |  |  |  |  |  |  |  |  |  |  | 9.86Aa | 9.52Aa | 7.72Aa | 7.23Aa | 8.58 |  |  |  |  |  |  |  |
| TA003858-0637 | 6A | A | 104 |  |  |  |  |  |  |  |  |  |  |  |  | 9.23Bb | 9.05Bb | 7.29Bb | 6.82Bb | 8.10 | 0.63 |  |  |  |  |  |  |
|  |  | G | 99 |  |  |  |  |  |  |  |  |  |  |  |  | 10.05Aa | 9.60Aa | 7.92Aa | 7.33Aa | 8.73 |  |  |  |  |  |  |  |
| TA005366-0788 | 6A | C | 172 |  |  |  |  |  |  |  |  |  |  |  |  | 9.54Ab | 9.27Aa | 7.53Ab | 7.01Ab | 8.34 | 0.41 |  |  |  |  |  |  |
|  |  | T | 33 |  |  |  |  |  |  |  |  |  |  |  |  | 10.11Aa | 9.65Aa | 7.80Aa | 7.44Aa | 8.75 |  |  |  |  |  |  |  |
| TA005690-1190 | 6A | A | 100 |  |  |  |  |  |  |  |  |  |  |  |  | 10.04Aa | 9.61Aa | 7.92Aa | 7.33Aa | 8.73 | 0.62 |  |  |  |  |  |  |
|  |  | G | 105 |  |  |  |  |  |  |  |  |  |  |  |  | 9.25Bb | 9.06Bb | 7.30Bb | 6.83Bb | 8.11 |  |  |  |  |  |  |  |

E1, Tai’an 2015; E2, Dezhou 2015; E3, Tai’an 2016; E4, Dezhou 2016; Difference, the phenotypic difference between alleles; A & B: Different capital letters indicate significant difference between alleles at one locus at *P* ≤0.01; a & b: Different lowercase letters indicate significant difference between alleles at one locus at *P* ≤0.05. L* value: the brightness index, a* value: the redness index, b* value: the yellowness index.

**Table S3** Candidate genes predication of loci significant associated with four colour traits

| **Marker** | **Chromosome** | **Candidate gene** | **Function** | **Biological process/Expression** | **Species** |
| --- | --- | --- | --- | --- | --- |
| Excalibur_c4709_576 | 2B | TraesCS2B01G243700 | [glycerol-3-phosphate O-acyltransferase activity](https://www.ebi.ac.uk/QuickGO/term/GO:0004366)  [protein self-association](https://www.ebi.ac.uk/QuickGO/term/GO:0043621) | [fatty acid biosynthetic process](https://www.ebi.ac.uk/QuickGO/term/GO:0006633)  [glycerol-3-phosphate metabolic process](https://www.ebi.ac.uk/QuickGO/term/GO:0006072)  [triglyceride biosynthetic process](https://www.ebi.ac.uk/QuickGO/term/GO:0019432) | Triticum aestivum (Wheat) |
|  |  |  | [glycerol-3-phosphate O-acyltransferase activity](https://www.ebi.ac.uk/QuickGO/term/GO:0004366)  [protein self-association](https://www.ebi.ac.uk/QuickGO/term/GO:0043621)  [sn-1-glycerol-3-phosphate C16:0-DCA-CoA acyl transferase activity](https://www.ebi.ac.uk/QuickGO/term/GO:0102420) | [diacylglycerol biosynthetic process](https://www.ebi.ac.uk/QuickGO/term/GO:0006651)  [fatty acid biosynthetic process](https://www.ebi.ac.uk/QuickGO/term/GO:0006633)  [glycerol-3-phosphate metabolic process](https://www.ebi.ac.uk/QuickGO/term/GO:0006072)  [lipid storage](https://www.ebi.ac.uk/QuickGO/term/GO:0019915)  [phospholipid biosynthetic process](https://www.ebi.ac.uk/QuickGO/term/GO:0008654)  [triglyceride biosynthetic process](https://www.ebi.ac.uk/QuickGO/term/GO:0019432) | Arabidopsis thaliana (Mouse-ear cress) |
| BS00000020_51 | 5D | TraesCS5D01G004300 | Pin-b | CM proteins would be involved in the cooking quality of pasta. | Triticum aestivum (Wheat) |
|  |  | Gsp-1D | an arabinogalactan peptide |  | Triticum aestivum (Wheat) |
| RAC875_c34446_396 | 6A | TraesCS6A01G062900LC.1 | NBS-LRR-like resistance protein | [carbohydrate metabolic process](https://www.ebi.ac.uk/QuickGO/term/GO:0005975) | Triticum aestivum (Wheat) |
| Tdurum_contig43646_147 | 1A | TraesCS1A01G065600 | [phosphatidic acid binding](https://www.ebi.ac.uk/QuickGO/term/GO:0070300) |  | Hordeum vulgare subsp. vulgare (Domesticated barley) |
|  |  |  | [lipoic acid binding](https://www.ebi.ac.uk/QuickGO/term/GO:0031405)  [zinc ion binding](https://www.ebi.ac.uk/QuickGO/term/GO:0008270) | [Fatty acid metabolism](https://www.uniprot.org/keywords/KW-0276)  [Lipid biosynthesis](https://www.uniprot.org/keywords/KW-0444)  [Lipid metabolism](https://www.uniprot.org/keywords/KW-0443) | Arabidopsis thaliana (Mouse-ear cress) |
| Ra_c105707_788 | 1A | TraesCS1A01G304400 | [2-succinyl-5-enolpyruvyl-6-hydroxy-3-cyclohexene-1-carboxylic-acid synthase activity](https://www.ebi.ac.uk/QuickGO/term/GO:0070204)  [thiamine pyrophosphate binding](https://www.ebi.ac.uk/QuickGO/term/GO:0030976)  [copper chaperone activity](https://www.ebi.ac.uk/QuickGO/term/GO:0016531)  [protein domain specific binding](https://www.ebi.ac.uk/QuickGO/term/GO:0019904) | [cellular amino acid catabolic process](https://www.ebi.ac.uk/QuickGO/term/GO:0009063)  [menaquinone biosynthetic process](https://www.ebi.ac.uk/QuickGO/term/GO:0009234)  [cellular copper ion homeostasis](https://www.ebi.ac.uk/QuickGO/term/GO:0006878)  [copper ion transport](https://www.ebi.ac.uk/QuickGO/term/GO:0006825)  [response to cadmium ion](https://www.ebi.ac.uk/QuickGO/term/GO:0046686) | Arabidopsis thaliana (Mouse-ear cress) |
| wsnp_RFL_Contig3802_4108582 | 2B | TraesCS2B01G245000 | [ionotropic glutamate receptor activity](https://www.ebi.ac.uk/QuickGO/term/GO:0004970) | Glutamate-gated receptor that probably acts as non-selective cation channel | Hordeum vulgare subsp. vulgare (Domesticated barley) |
| TA003589-0518 | 3A | TraesCS3A01G010000 | [thiol-dependent ubiquitin-specific protease activity](https://www.ebi.ac.uk/QuickGO/term/GO:0004843) | [protein deubiquitination](https://www.ebi.ac.uk/QuickGO/term/GO:0016579)  [ubiquitin-dependent protein catabolic process](https://www.ebi.ac.uk/QuickGO/term/GO:0006511) | Arabidopsis thaliana (Mouse-ear cress) |
|  |  | TraesCS3A01G010100 | [dephospho-CoA kinase activity](https://www.ebi.ac.uk/QuickGO/term/GO:0004140) | [coenzyme A biosynthetic process](https://www.ebi.ac.uk/QuickGO/term/GO:0015937) | Triticum aestivum (Wheat)  Arabidopsis thaliana (Mouse-ear cress) |
|  |  |  | [glucose-1-phosphate adenylyltransferase activity](https://www.ebi.ac.uk/QuickGO/term/GO:0008878) | [glycogen biosynthetic process](https://www.ebi.ac.uk/QuickGO/term/GO:0005978)  [starch metabolic process](https://www.ebi.ac.uk/QuickGO/term/GO:0005982) | Hordeum vulgare (Barley) |
|  |  |  |  |  |  |
| Jagger_c1888_277 | 1B | TraesCS1B01G011100 | [cyclosporin A binding](https://www.ebi.ac.uk/QuickGO/term/GO:0016018)  [peptidyl-prolyl cis-trans isomerase activity](https://www.ebi.ac.uk/QuickGO/term/GO:0003755)  [unfolded protein binding](https://www.ebi.ac.uk/QuickGO/term/GO:0051082) | [protein refolding](https://www.ebi.ac.uk/QuickGO/term/GO:0042026)  [protein folding](https://www.ebi.ac.uk/QuickGO/term/GO:0006457) | Triticum aestivum (Wheat)  Hordeum vulgare subsp. vulgare (Domesticated barley)  Arabidopsis thaliana (Mouse-ear cress) |
| Excalibur_c4152_1031 | 6A | TraesCS6A01G138600 | [serine-type carboxypeptidase activity](https://www.ebi.ac.uk/QuickGO/term/GO:0004185)  [tyrosine decarboxylase activity](https://www.ebi.ac.uk/QuickGO/term/GO:0004837) | [proteolysis involved in cellular protein catabolic process](https://www.ebi.ac.uk/QuickGO/term/GO:0051603)  [tyramine biosynthetic process](https://www.ebi.ac.uk/QuickGO/term/GO:1901695) | Triticum aestivum (Wheat) |
|  |  |  | [sialic acid transmembrane transporter activity](https://www.ebi.ac.uk/QuickGO/term/GO:0015136)  [UDP-N-acetylgalactosamine transmembrane transporter activity](https://www.ebi.ac.uk/QuickGO/term/GO:0005463) | [UDP-N-acetylgalactosamine transmembrane transport](https://www.ebi.ac.uk/QuickGO/term/GO:0015789)  [UDP-N-acetylglucosamine transmembrane transport](https://www.ebi.ac.uk/QuickGO/term/GO:1990569) | Arabidopsis thaliana (Mouse-ear cress) |
| Kukri_c65663_642 | 7A | TraesCS7A01G557700 | Leucine Rich Repeat family protein, expressed |  | Oryza sativa subsp. japonica (Rice) |
| RAC875_rep_c104674_867 | 7B | TraesCS7B01G482200 | [sucrose-phosphate synthase activity](https://www.ebi.ac.uk/QuickGO/term/GO:0046524)  [sucrose synthase activity](https://www.ebi.ac.uk/QuickGO/term/GO:0016157) | [sucrose metabolic process](https://www.ebi.ac.uk/QuickGO/term/GO:0005985) | Triticum aestivum (Wheat)  Arabidopsis thaliana (Mouse-ear cress)  Hordeum vulgare subsp. vulgare (Domesticated barley)  Oryza barthii |
| BS00064548_51 | 6A | TraesCS6A01G474500LC.1 | [4 iron, 4 sulfur cluster binding](https://www.ebi.ac.uk/QuickGO/term/GO:0051539)  [aconitate hydratase activity](https://www.ebi.ac.uk/QuickGO/term/GO:0003994) | [citrate metabolic process](https://www.ebi.ac.uk/QuickGO/term/GO:0006101)  [tricarboxylic acid cycle](https://www.ebi.ac.uk/QuickGO/term/GO:0006099) | Glycine max (Soybean) (Glycine hispida) |
| BS00027770_51 | 6B | TraesCS6B01G419200 | [fructose 1,6-bisphosphate 1-phosphatase activity](https://www.ebi.ac.uk/QuickGO/term/GO:0042132) | [carbohydrate metabolic process](https://www.ebi.ac.uk/QuickGO/term/GO:0005975) | Hordeum vulgare subsp. vulgare (Domesticated barley) |
|  |  |  | [endopeptidase activity](https://www.ebi.ac.uk/QuickGO/term/GO:0004175)  [threonine-type endopeptidase activity](https://www.ebi.ac.uk/QuickGO/term/GO:0004298) | [proteasomal protein catabolic process](https://www.ebi.ac.uk/QuickGO/term/GO:0010498)  [proteasomal ubiquitin-independent protein catabolic process](https://www.ebi.ac.uk/QuickGO/term/GO:0010499)  [proteasome-mediated ubiquitin-dependent protein catabolic process](https://www.ebi.ac.uk/QuickGO/term/GO:0043161) | Arabidopsis thaliana (Mouse-ear cress) |
|  |  |  | [dioxygenase activity](https://www.ebi.ac.uk/QuickGO/term/GO:0051213)  [jasmonic acid hydrolase](https://www.ebi.ac.uk/QuickGO/term/GO:0120091)  [metal ion binding](https://www.ebi.ac.uk/QuickGO/term/GO:0046872) | [flavonoid biosynthetic process](https://www.ebi.ac.uk/QuickGO/term/GO:0009813)  [regulation of jasmonic acid mediated signaling pathway](https://www.ebi.ac.uk/QuickGO/term/GO:2000022) | Arabidopsis thaliana (Mouse-ear cress) |
| BS00059383_51 | 3B | TraesCS3B01G037500 | [alcohol-forming fatty acyl-CoA reductase activity](https://www.ebi.ac.uk/QuickGO/term/GO:0102965)  [fatty-acyl-CoA reductase (alcohol-forming) activity](https://www.ebi.ac.uk/QuickGO/term/GO:0080019) | [lipid metabolic process](https://www.ebi.ac.uk/QuickGO/term/GO:0006629)  [long-chain fatty-acyl-CoA metabolic process](https://www.ebi.ac.uk/QuickGO/term/GO:0035336)  [suberin biosynthetic process](https://www.ebi.ac.uk/QuickGO/term/GO:0010345)  [wax biosynthetic process](https://www.ebi.ac.uk/QuickGO/term/GO:0010025) | Arabidopsis thaliana (Mouse-ear cress) |
|  |  |  | [serine-type peptidase activity](https://www.ebi.ac.uk/QuickGO/term/GO:0008236) |  | Hordeum vulgare subsp. vulgare |
| Kukri_c1214_2686 | 5B | TraesCS5B01G536600 | [glutamate synthase (ferredoxin) activity](https://www.ebi.ac.uk/QuickGO/term/GO:0016041) | [glutamate biosynthetic process](https://www.ebi.ac.uk/QuickGO/term/GO:0006537) | Arabidopsis thaliana (Mouse-ear cress) |
|  |  |  |  | [serine-type endopeptidase inhibitor activity](https://www.ebi.ac.uk/QuickGO/term/GO:0004867) | Aegilops tauschii（Tausch's goatgrass）（Aegilops squarrosa） |
| TA003858-0637 | 6A | TraesCS6A01G240400 | [4-hydroxy-tetrahydrodipicolinate reductase](https://www.ebi.ac.uk/QuickGO/term/GO:0008839) | [lysine biosynthetic process via diaminopimelate](https://www.ebi.ac.uk/QuickGO/term/GO:0009089) | Arabidopsis thaliana (Mouse-ear cress) |
|  |  | TraesCS6A01G240500 | [metallocarboxypeptidase activity](https://www.ebi.ac.uk/QuickGO/term/GO:0004181) | [peptide metabolic process](https://www.ebi.ac.uk/QuickGO/term/GO:0006518)  [protein processing](https://www.ebi.ac.uk/QuickGO/term/GO:0016485) | Arabidopsis thaliana (Mouse-ear cress) |
|  |  |  | [diacylglycerol kinase activity](https://www.ebi.ac.uk/QuickGO/term/GO:0004143)  [NAD+ kinase activity](https://www.ebi.ac.uk/QuickGO/term/GO:0003951) | [protein kinase C-activating G protein-coupled receptor signaling pathway](https://www.ebi.ac.uk/QuickGO/term/GO:0007205) | Triticum aestivum (Wheat) |
| TA005366-0788 | 6A | TraesCS6A01G240600 | [ATP binding](https://www.ebi.ac.uk/QuickGO/term/GO:0005524)  [metalloendopeptidase activity](https://www.ebi.ac.uk/QuickGO/term/GO:0004222) |  | Triticum aestivum (Wheat) |
|  |  |  | [omega-3 fatty acid desaturase activity](https://www.ebi.ac.uk/QuickGO/term/GO:0042389) | [unsaturated fatty acid biosynthetic process](https://www.ebi.ac.uk/QuickGO/term/GO:0006636) | Arabidopsis thaliana (Mouse-ear cress) |
| TA005690-1190 | 6A | TraesCS6A01G241200 | [hydroxymethylglutaryl-CoA lyase activity](https://www.ebi.ac.uk/QuickGO/term/GO:0004419)  [metal ion binding](https://www.ebi.ac.uk/QuickGO/term/GO:0046872) | [ketone body biosynthetic process](https://www.ebi.ac.uk/QuickGO/term/GO:0046951)  [leucine catabolic process](https://www.ebi.ac.uk/QuickGO/term/GO:0006552)  [lipid metabolic process](https://www.ebi.ac.uk/QuickGO/term/GO:0006629) | Arabidopsis thaliana (Mouse-ear cress) |

**Table S4** Predicted candidate genes for dough sheet color

| Marker | Chromosome | Candidate gene | Function | Biological process/Expression | Species |
| --- | --- | --- | --- | --- | --- |
| BS00065510_51 | 1D | TraesCS1D01G070200 | [ATP binding](https://www.ebi.ac.uk/QuickGO/term/GO:0005524)  [RNA binding](https://www.ebi.ac.uk/QuickGO/term/GO:0003723)  [RNA helicase activity](https://www.ebi.ac.uk/QuickGO/term/GO:0003724)  [ATP binding](https://www.ebi.ac.uk/QuickGO/term/GO:0005524)  [RNA binding](https://www.ebi.ac.uk/QuickGO/term/GO:0003723)  [RNA helicase activity](https://www.ebi.ac.uk/QuickGO/term/GO:0003724)  [ATP binding](https://www.ebi.ac.uk/QuickGO/term/GO:0005524)  [nucleic acid binding](https://www.ebi.ac.uk/QuickGO/term/GO:0003676) | [RNA catabolic process](https://www.ebi.ac.uk/QuickGO/term/GO:0006401)  [rRNA 3'-end processing](https://www.ebi.ac.uk/QuickGO/term/GO:0031125)  [RNA catabolic process](https://www.ebi.ac.uk/QuickGO/term/GO:0006401) | Oryza barthii  Aegilops tauschii（Tausch's goatgrass）（Aegilops squarrosa）  Triticum aestivum (Wheat) |
| BS00065722_51 | 1D | TraesCS1D01G070300 | [ATP binding](https://www.ebi.ac.uk/QuickGO/term/GO:0005524)  [RNA binding](https://www.ebi.ac.uk/QuickGO/term/GO:0003723)  [RNA helicase activity](https://www.ebi.ac.uk/QuickGO/term/GO:0003724) | [RNA catabolic process](https://www.ebi.ac.uk/QuickGO/term/GO:0006401)  [rRNA 3'-end processing](https://www.ebi.ac.uk/QuickGO/term/GO:0031125) | Oryza barthii |
| GENE-1258_171 | 2A | TraesCS2A01G593500 | [ATP binding](https://www.ebi.ac.uk/QuickGO/term/GO:0005524)  [Protein tyrosine kinase activity](https://www.ebi.ac.uk/QuickGO/term/GO:0004713)  [ATP binding](https://www.ebi.ac.uk/QuickGO/term/GO:0005524)  [Protein serine/threonine kinase activity](https://www.ebi.ac.uk/QuickGO/term/GO:0004674)  [ATP binding](https://www.ebi.ac.uk/QuickGO/term/GO:0005524)  [Protein kinase activity](https://www.ebi.ac.uk/QuickGO/term/GO:0004672) |  | Oryza barthii  Aegilops tauschii  Triticum aestivum (Wheat) |
| BS00099534_51 | 5A | TraesCS5A01G003800 | [catalytic activity](https://www.ebi.ac.uk/QuickGO/term/GO:0003824) |  | Triticum aestivum (Wheat) |
|  |  |  | [transferase activity, transferring glycosyl groups](https://www.ebi.ac.uk/QuickGO/term/GO:0016757) | [fucose metabolic process](https://www.ebi.ac.uk/QuickGO/term/GO:0006004) | Glycine max (Soybean) (Glycine hispida) |
|  |  |  | [protein kinase binding](https://www.ebi.ac.uk/QuickGO/term/GO:0019901)  [protein serine/threonine kinase activity](https://www.ebi.ac.uk/QuickGO/term/GO:0004674) | [protein phosphorylation](https://www.ebi.ac.uk/QuickGO/term/GO:0006468) | Arabidopsis thaliana (Mouse-ear cress) |
| BobWhite_c15802_72 | 6A | TraesCS6A01G024900 | [ATP binding](https://www.ebi.ac.uk/QuickGO/term/GO:0005524)  [nucleic acid binding](https://www.ebi.ac.uk/QuickGO/term/GO:0003676) |  | Triticum aestivum (Wheat) |
|  |  |  | [heme binding](https://www.ebi.ac.uk/QuickGO/term/GO:0020037)  [peroxidase activity](https://www.ebi.ac.uk/QuickGO/term/GO:0004601)  [adenine phosphoribosyltransferase activity](https://www.ebi.ac.uk/QuickGO/term/GO:0003999) | [response to oxidative stress](https://www.ebi.ac.uk/QuickGO/term/GO:0006979)  [adenine salvage](https://www.ebi.ac.uk/QuickGO/term/GO:0006168) | Glycine max (Soybean) (Glycine hispida) |
|  |  |  | [hydrolase activity, hydrolyzing O-glycosyl compounds](https://www.ebi.ac.uk/QuickGO/term/GO:0004553) | [carbohydrate metabolic process](https://www.ebi.ac.uk/QuickGO/term/GO:0005975) | Arabidopsis thaliana (Mouse-ear cress) |

**Table S5** Correlation coefficients(r) between colour traits of flour based on mean values across four environments

| **Traits** | **whiteness** | **L*** | **a*** | **b*** |
| --- | --- | --- | --- | --- |
| whiteness | 1 |  |  |  |
| L* | 0.605** | 1 |  |  |
| a* | 0.156* | 0.028 | 1 |  |
| b* | -0.901** | -0.108 | -0.204** | 1 |

L*, flour lightness; a*, flour redness; b*, flour yellowness

*and** significant at P=0.05 and P=0.01, respectively

**Table** **S6** Correlation coefficients(r) between dough sheet color based on mean values across four environments

| **Traits** | **L*** | **a*** | **b*** |
| --- | --- | --- | --- |
| L* | 1 |  |  |
| a* | -0.233** | 1 |  |
| b* | -0.479** | -0.406** | 1 |

L*, flour lightness; a*, flour redness; b*, flour yellowness

*and** significant at P=0.05 and P=0.01, respectively

**Table S7** Physical positions of SNP markers significantly associated with colour traits

| SNP marker | Physical position | SNP marker | Physical position |  |
| --- | --- | --- | --- | --- |
| Excalibur_c4709_576 | chr2B:247936325..247936921 (- strand) | Excalibur_c4152_1031 | chr6A:109983416..109988274 (+ strand) | |
| TA001505-1171 | chr2B:247925509..247935392 (+ strand) | wsnp_Ku_c38451_47086066 | chr6A:109983416..109988274 (+ strand) | |
| BS00000020_51 | chr5D:3609672..3610121 (+ strand) | Excalibur_c8883_214 | no |  |
| RAC875_c34446_396 | no | Excalibur_rep_c114255_439 | no |  |
| Tdurum_contig43646_147 | no | Kukri_c65663_642 | chr7A:730430495..730434493 (- strand) | |
| RAC875_c65431_351 | no | RAC875_rep_c104674_867 | chr7B:740032525..740041980 (- strand | |
| Excalibur_c12215_352 | no | Excalibur_rep_c92684_578 | chr7B:741242447..741246369 (- strand) | |
| Kukri_c3150_341 | chr1A:47823749..47827916 (- strand) | BobWhite_c10975_60 | chr7B:740038137..740041421 (- strand) | |
| BS00013227_51 | chr1A:48592864..48594183 (- strand) | Excalibur_c5938_1669 | chr7B:740038137..740041421 (- strand) | |
| BS00022870_51 | chr1A:48585417..48591127 (+ strand) | Excalibur_c5938_1703 | chr7B:740038137..740041421 (- strand) | |
| Kukri_c4900_2435 | no | Excalibur_c5938_371 | chr7B:740038137..740041421 (- strand) | |
| Tdurum_contig69753_513 | chr1A:47823749..47827916 (- strand) | Ex_c16529_304 | chr1A:494449856..494452574 (- strand) | |
| Ra_c105707_788 | chr1A:497516473..497521193 (+ strand) | BS00072153_51 | no |  |
| BS00089894_51 | chr1A:497516473..497521193 (+ strand) | BS00064548_51 | chr6A:521477191..521480678 (- strand) | |
| BS00093078_51 | chr1B:1201460..1203257 (+ strand) | Excalibur_c96915_247 | chr6B:707361771..707362719 (+ strand) | |
| Excalibur_c10657_1280 | chr1B:1248713..1255880 (+ strand) | BS00027770_51 | chr6B:690973003..690978947 (+ strand) | |
| Excalibur_c10657_796 | chr1B:1248713..1255880 (+ strand) | D_F5XZDLF02FKJFM_220 | chr7D:620213683..620215530 (+ strand) | |
| wsnp_BF200640B_Ta_2_1 | chr1B:627946124..627948201 (- strand) | Kukri_c57770_705 | no |  |
| RAC875_s109189_188 | chr2B:248140579..248150032 (+ strand) | BS00059383_51 | chr3B:18243443..18248804 (+ strand) | |
| wsnp_RFL_Contig3802_4108582 | chr2B:249207543..249213217 (- strand) | RAC875_c5834_235 | chr4A:737332328..737341420 (+ strand) | |
| TA003589-0518 | chr3A:8866086..8869744 (- strand) | Kukri_c1214_2686 | chr5B:692629200..692635084 (+ strand) | |
| Tdurum_contig10932_913 | chr7B:61557497..61564983 (+ strand) | Kukri_c1214_437 | chr5B:692629200..692635084 (+ strand) | |
| Kukri_c33486_128 | no | Kukri_c1214_544 | chr5B:692629200..692635084 (+ strand) | |
| GENE-4011_91 | chr6A:26974351..26978736 (- strand) | Kukri_c5228_1011 | chr5B:692629200..692635084 (+ strand) | |
| Jagger_c1888_277 | chr1B:5159471..5159817 (- strand) | TA003858-0637 | chr6A:451663192..451669168 (- strand) | |
| BS00072153_51 | no | TA005366-0788 | chr6A:451980227..451987318 (+ strand) | |
| BS00029348_51 | no | TA005690-1190 | chr6A:452637114..452641044 (- strand) | |
